# Causes of lower extremity weaknesses after posterior lumbar spine fusion surgery and therapeutic effects of active surgical exploration

**DOI:** 10.1186/s13018-020-01948-4

**Published:** 2020-09-22

**Authors:** Rui Wang, Chunde Li, Xiaodong Yi, Hailin Lu, Yu Wang, Hong Li

**Affiliations:** Department of Orthopaedics, Peking University First Hospital, No.8 of Xishiku Street, Xicheng District, Beijing, China

**Keywords:** Posterior lumbar spine fusion surgery, Neurological complications, Lower extremity weakness

## Abstract

**Background:**

This study was aimed at investigating the causes of lower extremity weaknesses after posterior lumbar spine fusion surgery and looking at subsequent treatment strategies.

**Methods:**

Patients who underwent posterior lumbar spine fusion surgery in the Peking University First Hospital between January 2009 and December 2018 were counted. Those who needed secondary surgery because of subsequent lower extremity weaknesses were selected. CT scans and MRIs were used to evaluate the reasons for weaknesses before secondary surgery. Muscle strength was evaluated after surgery.

**Results:**

Thirty patients (30/4078, 0.74%) required a secondary surgery because of lower extremity weaknesses after posterior lumbar spine fusion surgery. The main causes of weakness were (1) internal fixation malposition and loosening (11 patients, 36%), (2) epidural hematomas (9 patients, 30%), (3) insufficient decompression (5 patients, 17%), and (4) nerve root edemas (5 patients, 17%). Weakness occurred on average 2.9 days after surgery (1–9 days). Twenty-seven patients (90%) got improved muscle strength after their secondary surgery.

**Conclusions:**

Iatrogenic neurologic deficits and lower extremity weaknesses were rare complications after posterior lumbar spine fusion surgeries, but important to recognize and manage. The main causes of weakness were internal fixation malposition and loosening, epidural hematomas, insufficient decompression, or root edemas. There may be positive, therapeutic effects to subsequent, active surgical exploration.

## Background

Posterior lumbar fusions—including posterolateral fusions, posterior lumbar interbody fusions, and transforaminal lumbar fusions—have become the main surgical treatment options for various spinal disorders, such as spondylolisthesis, scoliosis, stenosis, instability, trauma, or tumors. Iatrogenic neurologic deficits after surgery are rare, but the most feared complications of spinal surgery. They can manifest as radiculopathies, lower extremity weaknesses, spinal cord compressions, or postoperative neuropathic pains. Several studies have reported that the prevalence of deficits ranges from 0.8 to 6.1% [1, 2]. Severe complications may cause permanent damage to the neurologic system, so it is important that neurologic complications are recognized and managed.

Iatrogenic neurologic deficits may occur via a number of routes. The most common way is due to the mechanical compression of nerve roots, the spinal cord, or the dural sac. They may occur via an expanding, space-occupying process such as a nerve root edema, an epidural hematoma, or via compressor instrumentation. Direct compressions can also occur when deformity corrective measures result in neural element compressions. Less commonly, distraction injuries to the spinal cord can occur from an overcorrection to the sagittal balance, or column shortening/lengthening maneuvers [3].

Lower extremity motor weaknesses are some of the most severe iatrogenic neurologic complications after spinal surgery. Secondary surgery or a prompt, surgical exploration is usually mandatory before permanent neurologic damage develops. This study investigated the causes of lower extremity weaknesses after posterior lumbar spine fusion surgery and the therapeutic effects of secondary surgery when treating this complication.

## Materials and methods

### Patient population

We counted the patients at our center that underwent posterior lumbar fusion surgery between January 2009 and December 2018. Patients who needed secondary surgery because of lower extremity weaknesses were selected. CT scans and MRIs evaluated the reasons for weaknesses before surgery, and after surgery, muscle strength was registered.

### Inclusion criteria

Patients were included if one or more of the following symptoms were present: (1) the muscle strength of their lower limbs had declined by more than three grades after posterior lumbar fusion surgery and had not improved with traditional methods of treatment (rest; intravenous infusions of mannitol and methylprednisolone), and (2) the muscle strength of their lower limbs had declined suddenly to grade 0 or 1 after fusion surgery.

### Exclusion criteria

Patients were excluded if the iatrogenic neurological deficits were ameliorated by traditional methods of treatment, or secondary surgery was needed because of other non-neurogenic factors, i.e., wound exudations, wound infections, or foreign body residues.

### Secondary surgery/exploration

After general anesthesia was induced, the initial incisions were made. There was surgical exploration of the mechanical compression of nerve roots, the spinal cord, and the dural sac before secondary surgery. All the potential compressions were removed, and drainage tubes were placed before the wound was closed in layers. Following closure, the drainage tubes were connected for negative suction drainage. The drainage tubes were kept unobstructed and only removed after the drainage volume was under 50 ml per day for 3 continuous days.

### Clinical evaluation

Causes of lower extremity motor weaknesses were evaluated according to the imaging results, i.e., the CT scans and MRIs, or exploration results during the secondary surgery. The muscle strength of patients was recorded and evaluated for 3 days and then 4 further days after surgery. The Wilcoxon-Mann-Whitney test was used to compare continuous, non-parametric variables, and the chi-square test was used to compare parametric categorical variables. Probability values < 0.05 were considered significant.

## Results

### Patient factors

Between 2009 and 2018, 4078 patients had posterior lumbar fusion surgeries at our center. Thirty patients (30/4078, 0.74%) needed secondary surgery, specifically because of lower extremity weaknesses. There were 12 men and 18 women with an average age of 62.9 years (62.9 ± 12.7 years). The most common indications that further surgery was required were where there were cases of lumbar herniated discs, spondylolisthesis, scoliosis, and lumbar stenosis (Table 1).

**Table 1:** Patients’ data

Variable	Mean ± SD or number
Patients	30
Males/females	12 (40%)/18 (60%)
Age	62.9 ± 12.7
Diagnosis	
Lumbar disc herniation	6
Stenosis	4
Lumbar disc herniation and stenosis	11
Scoliosis	7
Spondylolisthesis	2
Fusion segments	
2 segments	7
3 segments	11
4 segments	4
5 segments	1
> 5 segments	7

### Secondary surgical exploration outcomes

**Fig. 1:**
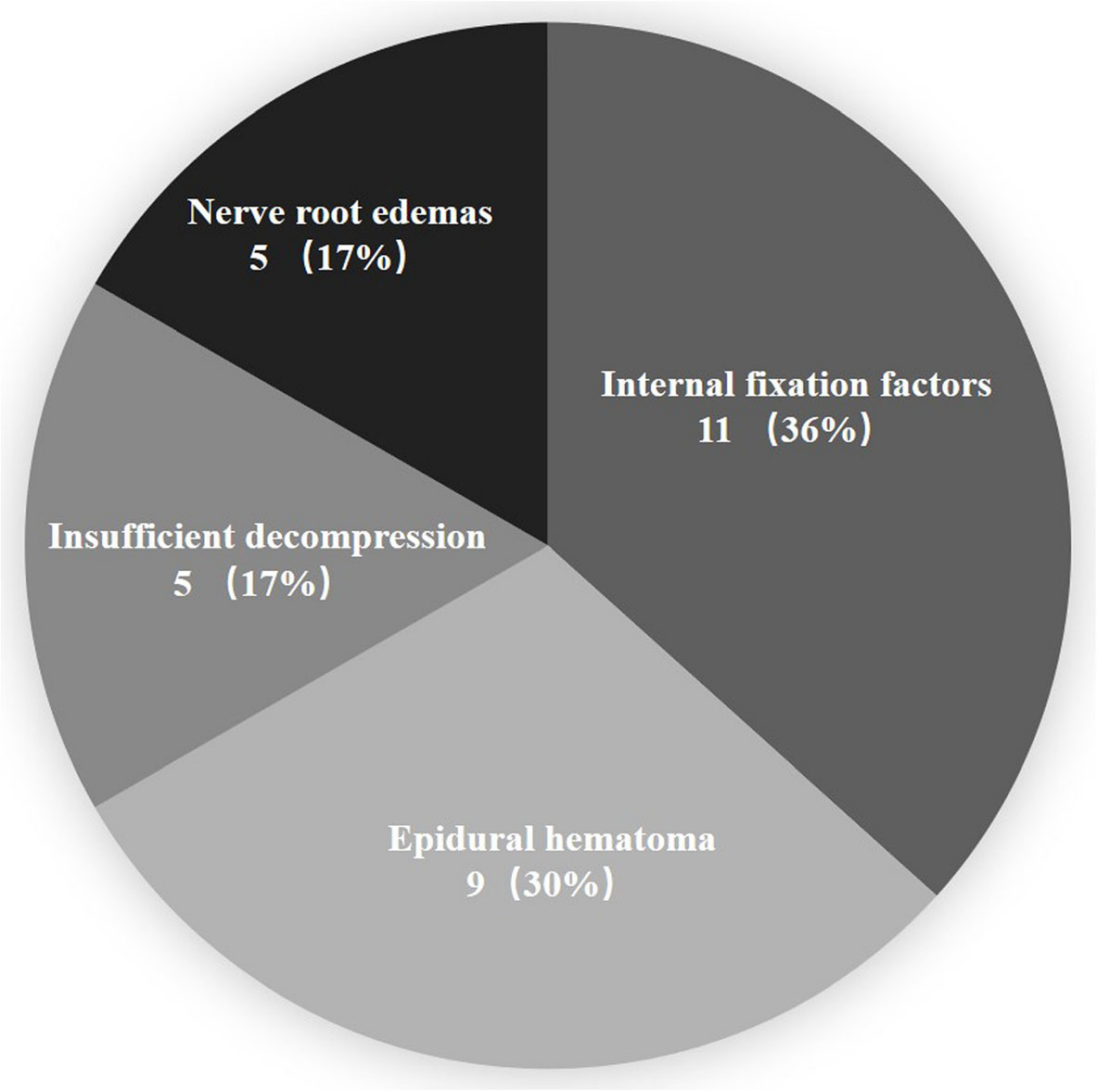
The main causes of lower extremity motor weaknesses after posterior lumbar fusion surgery

**Fig. 2:**
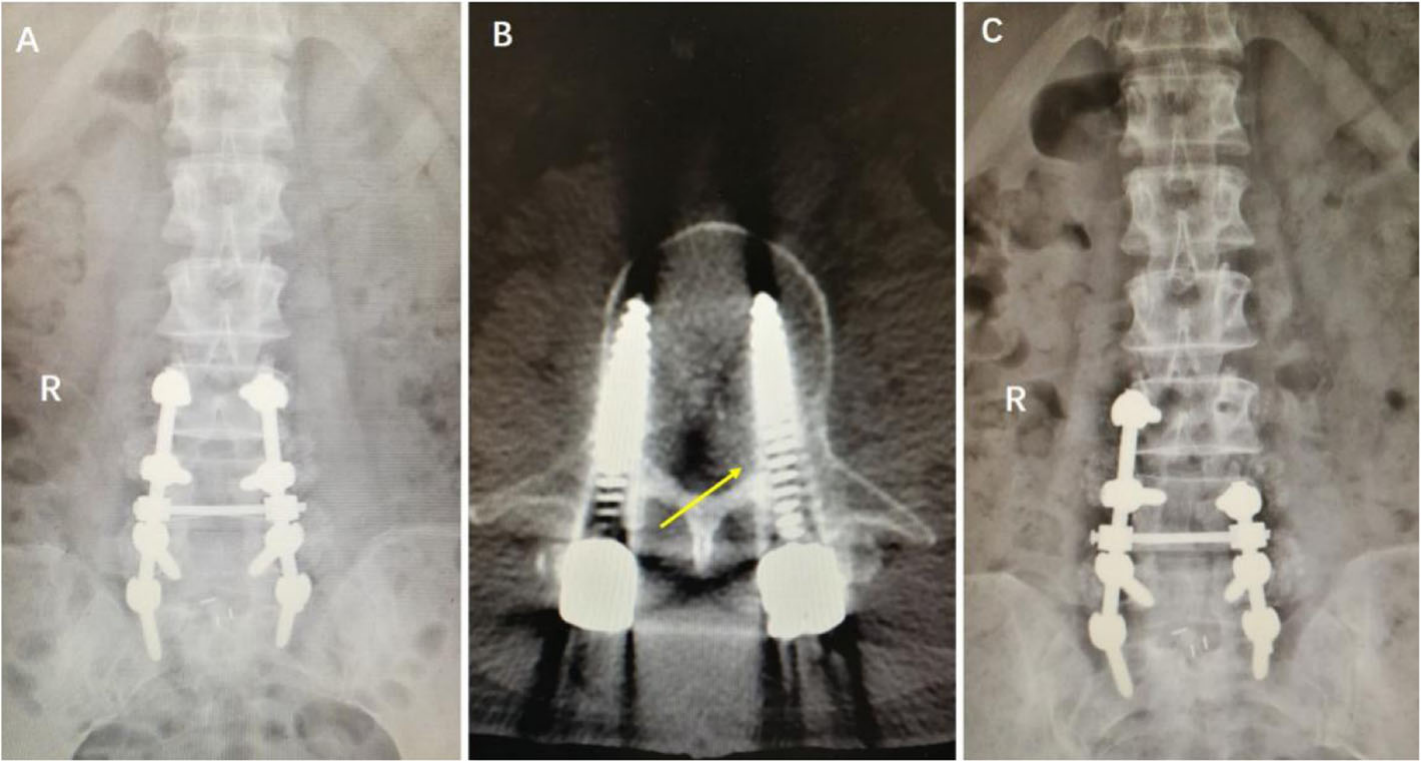
A 62-year-old female experienced pain in the front of her left thigh and had grade 1 hip flexion muscle strength 3 days after an L3–S1 PLIF. **a** Postoperative x-ray. **b** CT scan showed that the left L3 pedicle screw intruded the inner pedicle wall (arrow). **c** The left L3 pedicle screw was removed after reoperation. The patient’s symptoms were relieved after reoperation

**Fig. 3:**
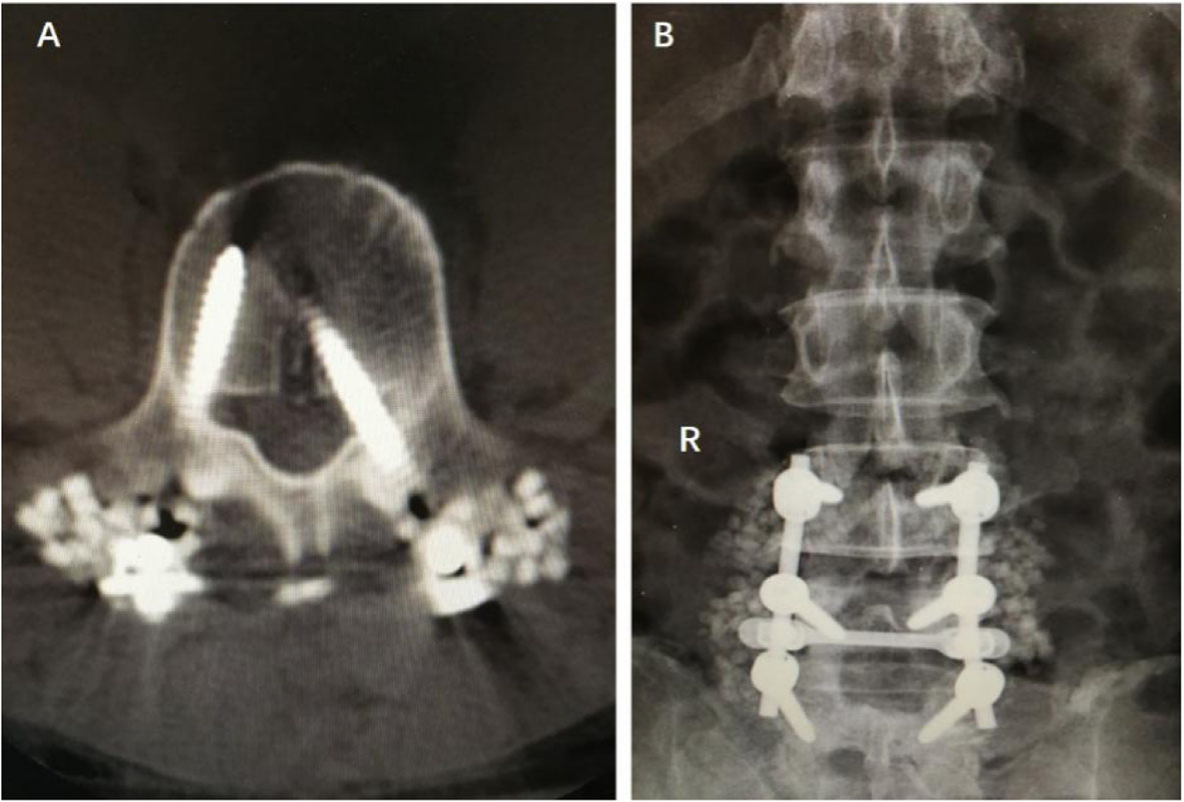
A 44-year-old male experienced numbness in his left dorso-phalangeal toe and had dorsiflexion and grade 2 muscle strength in his left ankle 1 day after an L4/5 discectomy and an L3–5 PLF. **a** CT scan showed that the left L4 pedicle screw had entered the spinal canal. **b** X-ray after the left L4 pedicle screw was adjusted during surgery. The patient’s symptoms (numbness, weakness) were relieved after reoperation

**Fig. 4:**
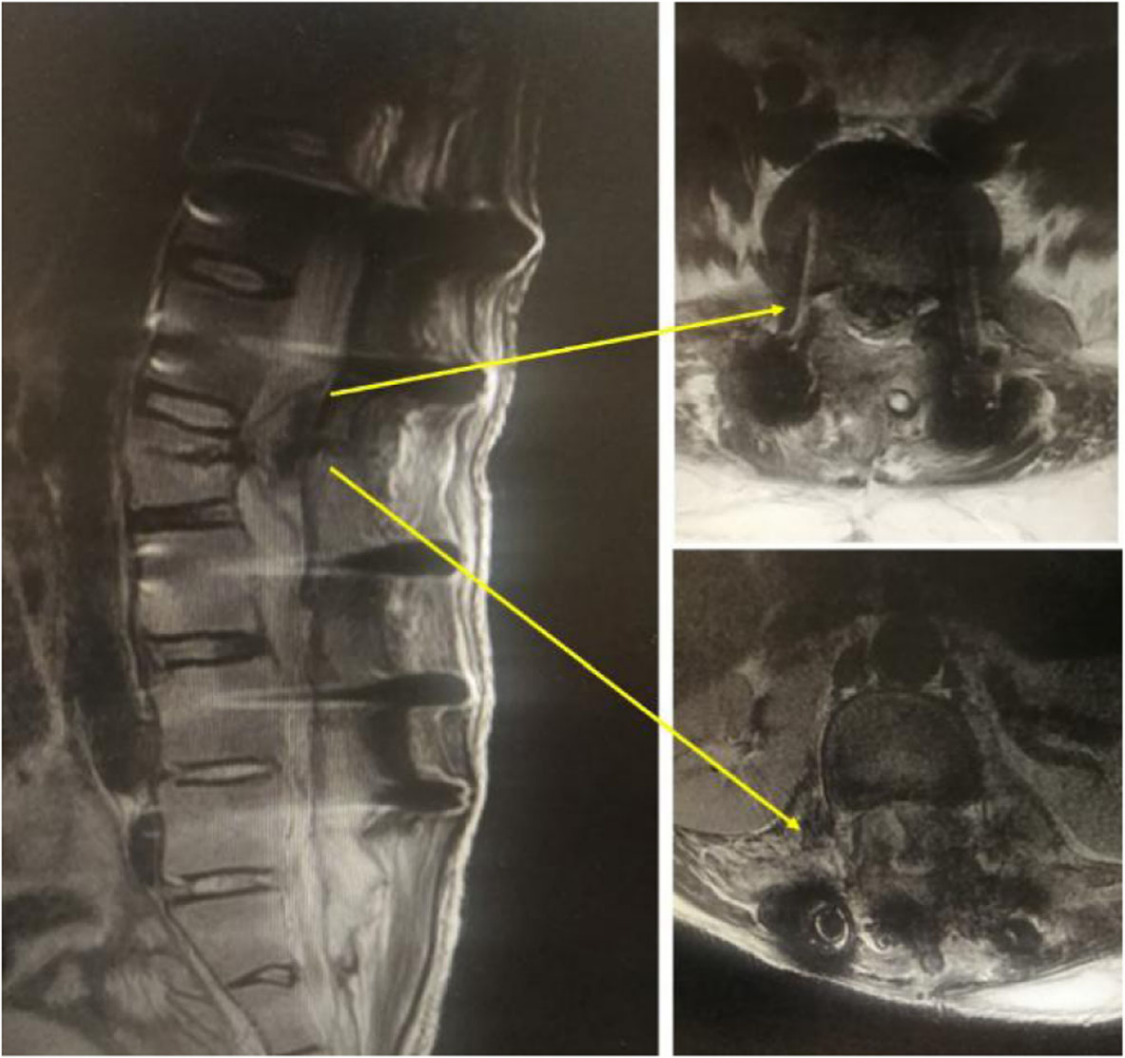
A 26-year-old male experienced numbness in his right lower extremity and had grade 1 hip flexion muscle strength a day after a PLF and an L2 pedicle subtraction osteotomy due to ankylosing spondylitis. The MRI showed an epidural hematoma behind the L2 vertebral body. The patient’s symptoms were relieved, and his muscle strength recovered after the hematoma was removed

According to the imaging and exploration results of secondary surgeries, the main causes of lower extremity weaknesses included (1) internal fixation malposition and loosening (11 patients, 36%), (2) epidural hematomas (9 patients, 30%), (3) insufficient decompressions (5 patients, 17%), and (4) nerve root edemas (5 patients, 17%) (Figs. 1, 2, 3, and 4). Out of the 11 patients who had internal fixation problems, eight needed revisiting because of malpositioning, two had loose pedicle screws, and one patient had an interbody fusion cage herniation. Out of the five patients with insufficient decompressions, two had asymptomatic complications linked to the ossification of ligamentum flavum and suffered compressions to the spinal cord during the correction of the sagittal balance. Three patients had lower extremity symptoms before fusion surgery, and although decompression procedures were performed, they complained of weaknesses on the other side of the lower extremities that had not exhibited before fusion surgery.

The average time that it took for post-surgery weaknesses to develop was 2.9 days (2.9 ± 1.7 days). Weaknesses caused by epidural hematomas happened much more quickly; the average time was 1.4 days (1.4 ± 0.5 days), whereas weaknesses after nerve root edemas took the longest average time to develop (4.8 ± 2.0 days) (Table 2).

**Table 2:** The average time for weakness to develop after surgery was due to different reasons

Causes of weakness	Average time after surgery (days)	***P*** value^**▲**^
Internal fixation factors	3.4 ± 1.2	0.238
Epidural hematomas	1.4 ± 0.5	0.026*
Insufficient decompressions	2.6 ± 1.1	0.079
Nerve root edemas	4.8 ± 2.0	

^**▲**^Compared to the “nerve root edemas” group

*"Epidural hematomas" group was significantly shorter compared to the "nerve root edemas" group

### Muscle strength outcomes

Out of the 30 patients assessed, 27 patients (90%) experienced immediate relief of their motor weakness symptoms after secondary surgery. Muscle strength in the lower extremities of these patients was recorded at grade 3 or 4 by the third day after surgery. By the seventh day, all patients were recorded as grade 4. Only three patients who had had epidural hematomas had lower extremity muscle strength (grade 2) by day 3, but they similarly were recorded as having grade 4 or normal muscle strength after secondary surgery.

## Discussion

Neurologic dysfunction or iatrogenic neurologic deficits after lumbar spine surgery are arguably the most severe complications of spinal surgery. They may result in neurological symptoms, such as radiculopathy, lower extremity weaknesses, postoperative neuropathic pains, or even short-term, permanent damage to the nervous system. Delamarter et al. [4] demonstrated in a study on dogs that when compressions to the spinal cord last for 6 h, there are no neurological recovery and progressive spinal cord necrosis. Other studies indicate that if patients with an acute spinal cord compression have had surgical decompressions within 8 h, their neurological functions will make good or partial recovery [5, 6]. A timely diagnosis and urgent management of neurologic complications are very important when helping patients recover from neurological deficits after lumbar spine surgeries. CT scans and MRIs are mainstream examinations that help in the diagnosis of neurological deficits.

Neurological deficits after lumbar spine surgeries are rare complications, and their occurrence rate varies widely in different studies. There are also patient variables, including age, general level of health, and previous surgical procedures/lumbar fusions. Kamerlink et al. [7] found that hyperkyphotic patients undergoing anteroposterior deformity corrections were at a relatively higher risk of postoperative neurological deficits. This is due to a disruption of blood flow to the thoracic spinal cord through segmented arterial feeders from the aorta. Carreon et al. [1] demonstrated in a retrospective study that the occurrence rate of perioperative neurological deficits was 2% (2/98) after posterior lumbar decompression and arthrodesis in older adults (≥ 65 years of age). Daubs et al. [8] found in another retrospective study that the rate of neurologic deficits in patients ≥ 60 years of age who underwent major spinal deformity surgeries and required a minimum level 5 arthrodesis procedure was 8.7% (4/46). Another study by Bydon et al. into 500 lumbar discectomies found a 2.61% rate of postoperative weakness [9]. A recent meta-analysis study by Ghobrial et al. [3] showed that 37 out of 2052 (1.9%) patients had a neurologic injury after posterior decompressions and fusions. Disparities between these studies are as to be expected, due to statistical and operative factors, variations on the definitions of postoperative neurological deficits, inclusion and exclusion criteria, surgical complexities, and the number of levels instrumented. In this study, we found that the incidence rate of lower extremity weaknesses was 0.74% (30/4078) after posterior lumbar spine fusion surgeries. The exclusion criterion for the present study was where there were cases of symptoms easing after neurological deficits, due to traditional methods of treatment. We should be aware, of course, that there is a greater rate of morbidity where there are neurological deficit complications.

Our study demonstrated that the malposition or loosening of fixations was one of the most common causes of weakness after spinal fusion surgery. Ghobrial et al. [3] showed that in a study of 37 patients suffering neurological injury after spinal fusion surgery, a malposition of screws resulted in 11 injuries and 9 patients were affected by the placement of instrumentation [10–13]. Lee et al. [14] reported in their study that CT scans detected a rate of screw malposition as 3.9%. Numerous studies show that the use of image-guided technologies to identify pedicle screw placements could significantly decrease the pedicle breach rate during a procedure [15, 16]. Neuromonitoring and SSEPs enable the earlier detection of potential injuries and significantly limit neurological deficits [3].

Our study found that epidural hematomas were one of the most common causes of weaknesses. In addition, the weaknesses caused by epidural hematomas occurred within the shortest average time after surgery: 1.4 days. There should be a considerable diagnosis for acute epidural hematomas if neurological deficits are found shortly after surgery. Kou et al. [6] found that patients requiring multilevel lumbar procedures, especially those with preoperative coagulopathy, were at a significantly higher risk of developing a postoperative epidural hematoma. In our study, one patient underwent L3–S1 fusion procedures and suffered a preoperative congenital deficiency of coagulation factor XIII. Lower extremity motor weaknesses occurred the day after surgery, and an acute epidural hematoma was found on an MRI.

Other minor causes of lower extremity weaknesses after spinal fusion surgery were insufficient decompressions during surgery and nerve root edemas. In our study, we found three patients with unilateral lower extremity symptoms prior to surgery. Decompression procedures were performed, but patients complained of motor weaknesses on the other side of the lower extremity. They had had no symptoms before the fusion surgery. The reason for this is still unknown. A possible reason is that there has been a relative shift of the vertebrae due to internal instrumentation during surgery. This may have resulted in the stimulation and/or compression of nerve roots. In addition, tractions of nerve roots during surgery and a congestive reaction after surgery can lead to an edema of nerve roots, thus resulting in radiculopathy, neuropathic pain, and motor weakness. Several studies demonstrated that steroid administration could modify the edema and the inflammatory response of nerve roots in patients, without increased incidence of postoperative infection or suture failures [17, 18]. In our surgical center, an intravenous infusion of methylprednisolone was routine after lumbar decompressions and fusion surgeries. We found that only five patients needed secondary surgery because of a postoperative nerve root edema.

There are several limitations in this study. First, the study was retrospective. Secondly, patients were collected from a single institute and the number of patients was relatively small because of the low number of neurological complications after spinal surgery. To resolve these limitations, a prospective multi-institutional study is suggested.

## Conclusions

In summary, our study reveals that iatrogenic neurological deficits and lower extremity weaknesses after posterior lumbar spine fusion surgeries were rare complications, but important to recognize and manage. The main cause of weaknesses was internal fixation problems, epidural hematomas, insufficient decompressions, and nerve root edemas. Active, surgical exploration may lead to positive, therapeutic effects.

## Data Availability

Not applicable.
